# Botulinum toxin A for the treatment of strabismus in children with neurological impairment

**DOI:** 10.5935/0004-2749.2021-0401

**Published:** 2023-03-08

**Authors:** Betul Tugcu, Bilge Araz-Ersan, Seyhan B. Özkan

**Affiliations:** 1 Department of Ophthalmology, Bezmi Alem Vakif University, Istanbul, Turkey; 2 Department of Ophthalmology, Şişli Hamidiye Etfal Education and Research Hospital, Istanbul, Turkey; 3 Private practice, Aydın, Turkey

**Keywords:** Strabismus, Botulinum toxin, Neurological mani-festations, Nervous system diseases, Cerebral palsy, Hydrocephalus, Children, Estrabismo, Toxinas botulínicas, Manifestações neurológicas, Doenças do sistema nervoso, Paralisia cerebral, Hodrocefalia, Criança

## Abstract

**Purposes:**

To assess the efficacy of botulinum toxin A injection in the treatment of strabismus in patients with neurological impairment and evaluate the factors associated with treatment success.

**Methods:**

The study included 50 patients with strabismus and neurological impairment. In all children, botulinum toxin injection was performed into the appropriate extraocular muscle. The relationship between demographic features, clinical characteristics, and treatment success were analyzed.

**Results:**

In the study group, 34 patients had esotropia, and 16 patients had exotropia. As neurological problems, 36 patients had cerebral palsy, and 14 had hydrocephalus. The average follow-up period was 15.3 ± 7.3 months. The mean number of injections was 1.4 ± 0.6. The mean angle of deviation was 42.5 ± 13.2 PD before the treatment, which decreased to 12.8 ± 11.9 PD after the treatment. Successful motor alignment (orthotropia within 10 PD) was achieved in 60% of the patients. Binary logistic regression analysis revealed that esotropic misalignment and shorter duration of strabismus was significantly associated with treatment success in the study group. Patients with esotropia and lower angles of misalignment were more likely to be treated with a single injection.

**Conclusion:**

The use of botulinum toxin A for the treatment of strabismus in children with neurological impairment is a good alternative to conventional surgical therapy with a lower risk of overcorrection. The treatment outcome is better in esodeviations and shorter duration of strabismus, implying an advantage of early treatment.

## Abstract (pt)

**Objetivos:**

Avaliar a eficácia do uso de toxina bo-tulínica tipo A no tratamento do estrabismo em pacientes com comprometimento neurológico e avaliar os fatores associados ao sucesso do tratamento.

**Métodos:**

Cinquenta pacientes com estrabismo e comprometimento neurológico foram incluídos no estudo. Em todas as crianças, a toxina botulínica tipo A foi injetada no músculo extraocular apropriado. A relação entre características demográficas, características clínicas e o sucesso do tratamento foram analisadas.

**Resultados:**

No grupo de estudo, 34 pacientes tiveram esotropia e 16 pacientes tiveram exotropia, sendo trinta e seis pacientes com paralisia cerebral e 14 pacientes com hidrocefalia. O tempo médio de acompanhamento foi de 15,3 ± 7,3 meses. O número médio de aplicações foi de 1,4 ± 0,6. O ângulo de desvio médio foi de 42,5 ± 13,2 DP antes do tratamento e diminuiu para 12,8 ± 11,9 DP após o tratamento. Alinhamento motor bem sucedido (ortotropia dentro de 10 DP) foi alcançado em 60% dos pacientes. A análise de regressão logística binária revelou que o desalinhamento esotrópico e uma menor duração do estrabismo foram significativamente associados ao sucesso do tratamento no grupo de estudo. Pacientes esotrópicos com ângulos de desalinhamento menores são mais propensos a serem tratados com uma única aplicação.

**Conclusão:**

O uso da toxina botulínica tipo A para o tratamento de estrabismo em crianças com comprometimento neurológico é uma boa alternativa para a terapia cirúrgica convencional com menor risco de hipercorreção. O resultado do tratamento é melhor em exodesvios e em pacientes com estrabismo de menor duração, implicando em vantagem para o tratamento precoce.

## INTRODUCTION

Strabismus is a relatively common ocular disorder among children with neurological impairment, which affects >50% of this patient population^(1)^. In these children, the primary concern of the treatment of strabismus is the less predictable surgical results. Previous studies have suggested that poor patient cooperation, binocular dysfunction, variability of the angle of deviation, and lack of a standard surgical dose-effect relationship may negatively affect the surgical outcomes^(2^-^4)^. Further surgeries are often required to optimize eye alignment. However, multiple strabismus surgeries further increase the unpredictability of the results because of additional postoperative adhesions and tissue scarring.

Another significant problem in the treatment of these patients is the existence of associated comorbidities, which potentially increase the risk of perioperative morbidity and mortality related to general anesthesia. Thus, treatment tended to be postponed. However, this concept has been challenged by the importance of earlier eye alignment for visual, behavioral, and motor development in children with neurological impairment^(2)^.

To avoid the risks of surgical treatment and full general anesthesia, botulinum toxin type A (BT-A) injection has been used as an alternative for the treatment of strabismus. Thus, this study aimed to assess the efficacy of BT-A injection for the treatment of strabismus in patients with neurological impairment and analyze the factors affecting the success rate.

## METHODS

This noncomparative, retrospective, interventional case series was conducted in consecutive patients at the Pediatric Ophthalmology Department of Bezmi Alem Vakıf University (BT) and patients from a private clinic (SBÖ). The study was approved by the Institutional Review Board and followed the tenets of the Declaration of Helsinki for research involving human subjects. Informed consent was obtained from all parents of the patients.

All children with strabismus and with accompanying neurological problems who received BT-A injection were identified. The exclusion criteria were as follows: positive forced duction test exceeding + 1, presence of vertical deviations, coexistence of any other ophthalmic pathologies, and any history of ocular surgery.

All patients underwent full ophthalmologic examinations, including cycloplegic refraction and ocular motility examination. The amount of deviation was measured by either alternate prism cover test or Krimsky test depending on the degree of the child’s cooperation. All clinically significant refractive errors were corrected with glasses, and amblyopia was treated before the intervention until the deviation reached an alternating pattern. All ocular alignment measurements and all BT-A injections were performed by experienced ophthalmologists. Three measurements or more were obtained preoperatively, with at least two consecutive comparable measurements before the surgery.

Patients with no reduction in the angle of deviation were defined as nonresponders, and surgical intervention was considered. Patients with a decrease in the angle of deviation at least 30% of the preinjection deviation after 3 months were defined as partial responders, and reinjection was offered. The final angle of deviation was used for the statistical analysis. Successful motor alignment was defined as the deviation within 10 DP.

All children received bilateral BT-A injections as a quick procedure under general anesthesia provided by either a facial mask or ketamine anesthesia without intubation. If the angle of deviation were <40 and >40 DP, the dosages were 2.5 and 5 units of BT-A per muscle, respectively. For patients who required more than one injection, the same dose was used. A 27-gauge needle was inserted into the medial or lateral rectus muscle without electromyographic (EMG) guidance using Mendonça’s forceps or by monopolar needle electrode under EMG guidance.

All patients were followed for at least 6 months after the first injection (range, 6-36 months; mean, 15.3 ± 7.3 months). They were examined at 15 days, 3 months, 6 months, and every 6 months thereafter. The follow-up visits include the assessment of the angle of deviation, limitation in ocular motility, and ptosis.

Data were analyzed using SPSS Statistics for Windows version 17.0 (SPSS Inc., Chicago, IL, USA). Descriptive data are reported as mean ± standard deviation and percentage. Fisher’s exact test was used to compare proportions. Variables associated with treatment success were determined. The univariate analysis included sex, misalignment type, refractive error, neurological disorder, age, misalignment duration, age at misalignment initiation, number of injections, angle of deviation before injection, ptosis, vertical deviation, variability, latent nystagmus, infantile onset, retinopathy of prematurity (ROP), and preterm birth history as independent parameters, and the overall treatment success and success with a single BT-A injection were included as dependent parameters. Stepwise multivariate logistic regression analysis was performed to investigate the independent associations of the overall treatment success and success with a single BT-A injection. Odds ratios (OR) and 95% confidence intervals (CI) were applied to the analysis. A p-value of <0.05 was considered significant.

## RESULTS

The retrospective chart review identified 103 patients with varying severity of brain pathologies. Of these patients, BT-A injection was offered to 64 patients, accepted by 59; however, 9 of these were lost to follow-up. The demographic and clinical characteristics of the 50 patients included in the study are outlined in table 1. The mean pre-injection angle of deviation was 42.5 ± 13.2 (range, 25-90) DP, and at the final visit, the mean angle of deviation decreased to 12.8 ± 11.9 (range, 0-45) DP after the treatment. Successful motor alignment (orthotropia within 10 DP) was achieved in 60% of the patients. The success rate was 73.5% in the esotropic group and 31.3% in the exotropic group. The clinical characteristics of the esotropic and exotropic groups are summarized in table 2. The BT-A treatment was found to be significantly more effective in the esotropic group (p=0.006).

**Table 1:** Demographic and clinical characteristics of patients with esotropia and exotropia

Patient characteristics		
Sex	Female/male	29/21
Mean age (months)	Mean± SD Range	41.8 ± 28.9 6-132
Duration of misalignment (months)	Mean± SD Range	34 ± 27.3 4-108
Mean follow-up period (months)	Mean± SD Range	15.3 ± 7.3 6-36
Type of misalignment	Esotropia /exotropia	34/16
Brain pathology	Cerebral palsy/hydrocephalus	36/14
Refractive error (mean spherical equivalent) (diopters)	Mean± SD Range	2.6 ± 1.7 -2.50-7.50
Refractive error OD	Mean ± SD Range	2.6 ± 1.8 -4.00-7.50
Refractive error OS	Mean ± SD Range	2.7 ± 1.7 -1.375-7.50
Latent nystagmus		9
Infantile onset		33
Preterm birth history		19

SD= standard deviation.

**Table 2:** Clinical characteristics of patients with esotropia and exotropia

	Mean age (months) (min-max)	Duration of misalignment (months) (min-max)	Age at initiation of misalignment (months) (min-max)	Follow-up period (months) (min-max)	Neurological disorder	PreBTX deviation (pd)	Final visit deviation (pd)	Injection number	Ptosis	Success rate with a single injection	Success rate
TOTAL (50)	41.8 ± 28.9 (6-132)	34 ± 27.3 (4-108)	8.1 ± 12.7 (1-88)	15.3 ± 7.3 (6-36)	36 CP/14 HC	42.5 ± 13.2 (25-90)	12.8 ± 11.9 (0-45)	1.4 ± 0.6 (1-3)	15	44%	60%
ET (34)	36.7 (6-110)	29.3 (4-105)	7.6 (1-88)	14.4 (6-36)	21 CP/13 HC	41.513.85 (25-90)	10 ± 9.99 (0-45)	1.3 ± 0.5 (1-3)	12	55.9%	73.5%
XT (16)	52.6 (8-132)	44.1 (4-108)	9.1 (1-24)	17 (6-29)	15 CP/1 HC	44.7 ± 11.9 (30-80)	18.6 ± 13.7 (4-45)	1.56 ± 0.63 (1-3)	3	18.7%	31.3%
Pvalue (ET/XT)	0.069	0.073	0.089	0.252	**0.021**	0.186	**0.018**	0.105	0.328	**0.014**	**0.004**

CP= cerebral palsy; ET= esotropia; HC= hydrocephaly; XT= exotropia.

The mean number of injections was 1.4 ± 0.6. Because of residual deviation, reinjection was required in 17 (34%) patients, 15 (30%) received two injections, and 2 (4%) received three injections. The success rates with a single injection were 55.9% and 18.7% in the esotropic and exotropic groups, respectively. A single BT-A injection was significantly more effective in the esotropic group (p=0.017).

Of the 50 patients, successful alignment was achieved in 22 with a single injection (44%). The mean angle of deviation in patients who were successfully treated with a single injection (n=22) was 35.9 ± 6.1 (25-50) PD. In those who required second (n=7) and third (n=1) injections, the mean angles of deviation were 51.4 ± 19.5 (30-90) PD and 50 PD, respectively. Strabismus surgery was performed in 5 (4 with exotropia and 1 with esotropia) of the 11 patients who did not respond to BT-A injections. A successful outcome was obtained in all of these patients. The remaining six nonresponders refused the surgical treatment.

Vertical deviation related to BT-A injection was identified in 3 (6%) patients. Mild ptosis was observed in 14 (28%) patients, and moderate ptosis in 1 (2%) patient, which was related to the leakage of BT-A into the levator palpebra superioris muscle. Complete resolution of ptosis was noted in all patients at the third month of follow-up, and none of them required occlusion therapy. A temporary overcorrection of deviation was observed in 16 patients with esotropia (32%) and one patient with exotropia (2%). The overcorrection period lasted 8-12 weeks, and stable orthotropia was achieved subsequently. No anesthesiaor surgery-related adverse effects were observed in any of the patients. Duction deficit was not observed in any of the patients in the late post-injection period. Permanent consecutive deviation did not develop in any of the patients.

The univariate analysis showed that esotropic misalignment, hydrocephaly (HC), younger age, and shorter duration of misalignment were significantly associated with a higher overall treatment success. The binary logistic regression analysis including these variables revealed that esotropic misalignment and shorter duration of misalignment were significantly associated with treatment success. The univariate analysis demonstrated that esotropic misalignment and lower angle of deviation before injection were significantly associated with treatment success with a single BT-A injection. The binary logistic regression analysis including these variables revealed that successful treatment with a single BT-A injection was associated with esotropic misalignment and lower angle of deviation before injection. The results of the univariate and multivariate logistic regression analyses for overall treatment success and treatment success with a single injection are outlined in table 3.

**Table 3:** Univariate and multivariate logistic regression analyses for the overall successs and success with a single injection of botulinum toxin A

	Success						Success with a single injection					
	Univariate analysis			Multivariate analysis			Univariate analysis			Multivariate analysis		
	OR	95% CI	p-value	OR	95% CI	p-value	OR	95% CI	p-value	OR	95% CI	p-value
Sex (Female)	0.872	0.276-2.752	0.815				1.083	0.349-3.362	0.890			
Type of misalignment (esotropia)	6.111	1.660-22.493	**0.006**	5.127	1.248-21.059	**0,023**	5.489	1.318-22.851	**0.019**	4.986	1.069-23.262	**0.041**
Refractive error	1.036	0.745-1.441	0.833				1.333	0.926-1.919	0.123			
Neurological disorder (CP)	0.167	0.033-0.853	**0.032**				0.477	0.136-1.670	0.247			
Age	0.973	0.950-0.996	**0.02**				0.989	0.969-1.010	0.304			
Duration of misalignment	0.963	0.938-09.989	**0.006**	0.965	0.938-0.994	**0,016**	0.979	0.955-1.003	0.084			
Age at initiation of misalignment	1.024	0.958-1.095	0.483				1.054	0.961-1.155	0.264			
Injection number	0.533	0.193-1.474	0.225				-	-	-			
Angle of deviation before injection	0.963	0.918-1.010	0.118				0.878	0.801-0.961	**0.005**	0.885	0.808-0.970	**0.009**
Ptosis	2.316	0.616-8.700	0.214				1.263	0.370-4.315	0.709			
Early overcorrection	0.327	0.088-1.214	0.095				0.400	0.121-1.326	0.134			
Vertical deviation	0.310	0.026-3.674	0.353				0.619	0.052-7.307	0.703			
Latent nistagmus	0.462	0.107-1.988	0.299				0.300	0.056-1.620	0.162			
Infantile onset	1.077	0.327-3.546	0.903				0.578	0.177-1.882	0.363			
ROP	2.111	0.204-21.873	0.531				1.300	0.168-10.046	0.801			
Preterm birth history	1.784	0.538-5.914	0.344				1.759	0.554-5.582	0.338			

CP= cerebral palsy; ROP= retinopathy of prematurity.

## DISCUSSION

Previous studies evaluating the surgical treatment outcomes of strabismus in children with neurological impairment revealed a success rate ranging from 37.5% to 66%^(2^-^5)^. In the present study, the success rate was 60%. In reviewing the literature on strabismus, only a few studies have evaluated the efficacy of BT-A for the treatment of strabismus in children with neurological impairment^(6^-^11)^. The characteristics of patients and results of BT-A injection in these studies are outlined in table 4. The success rates of these studies were between 31% and 72%^(6^-^11)^. Three studies included patients with esotropia^(7^,^8^,^11)^, and three enrolled patients with esotropia and exotropia^(6^,^9^,^10)^. In a recent study, Mangan et al. included patients with esotropia and neurological disorders and/or prematurity and reported 51.7% success rate only for the whole study group (34 patients with neurological impairment and 22 patients born preterm)^(12)^. However, the success rate for the neurologically impaired subgroup was not given separately, which made it impossible to make a comparison with the results of our series because neurological problems are seen in only 14.7% of the preterm group^(13)^.

**Table 4:** Results of botulinum toxin injection in patients with neurological disorders in comparison of previous studies

Study	Year	Case	Type of misalignment	Sample size	Age (mean±SD)	Angle of deviation (mean ± SD) PD	Dose	Duration of follow-up	Average number of injection	Success rate with a single injection (%)	Success rate (%)	Persistent consecutive strabismus (%)
Moguel ^(13)^	2004	Paroxysmal diseases(11) CP (7) others (12)	ET (15) XT (15)	30	3.5 years	NA	2.5-10 IU Botulinum toxin type A	12.7 2.3 mo	1.7	43.3	ET 69.2 XT 46	0
Cronemberger^(12)^	2006	CP	ET (17) XT (7)	24	60.4 (6-156) mo	ET 34.4 (25-45) XT 32.1 (20-45)	4 IU Botox^TM^	24 mo	1.3	ET 47.1 XT 0	ET 47.1 XT 11.1	0
Hauville**r**^(14)^	2007	HC(8) CP(2) others (15)	ET	25	26.4 (9-76) mo	35 (20-60)	2.5-3.75 IU Botox^TM^	29 (6-59) mo	1.5	36	72	0
Arroyo-Yllanes^(11)^	2009	CP	ET	32	16.8 (5-60)	39.12	2.5-10 IU Botulinum toxin	1.4 years (6 mo-3.5 years)	1.6	NA	31.2	25
Segura-Rangel ^(9)^	2011	CP	ET (21) XT (24)	45	7 (1-18) years	ET 35 (18-60) XT 39 (20-60)	5-12.5 IU Botulinum toxin type A	≥1 year	1-3	ET 23 XT 4.1	ET 38.1 XT 16.6	9.5
Ameri^(10)^	2014	CP	ET	44	47.56 mo (5-124)	52.27 (25-123)	7IU Novotox^TM^	12-24 mo	1.3	NA	61.4	13.63
This study	2019	CP (36) HC (14)	ET (34) XT (16)	50	41.8 (6-132)	42.5 ± 13.2 (25-90)	5-10 IU Botox^TM^	15.3 (6-36) mo	1.4	44	60	0

ET= esotropia; XT= exotropia; CP= cerebral palsy; HC= hydrocephaly; NA= not available; mo= months.

Primarily, this study sought to determine whether BT-A injection could be preferred as an initial treatment in patients with strabismus and neurological disorders. Although a comparative study was not performed, according to these results, BT-A injection may have a comparable success rate to surgery in patients with strabismus and neurological impairment and a much lower risk of consecutive deviation. The rate of consecutive strabismus was reported up to 50% after surgical treatment, and reoperation was required in 23% of the patients^(2^,^3^,^5)^. In the evaluation of possible clinical risk factors for the development of consecutive strabismus after surgical treatment, accompanying neurological disease was found to be a significant factor^(14)^. Non-resolving consecutive strabismus following BT-A injection was reported in 1%-5% of otherwise healthy patients^(15)^. The most important risk factor for non-resolving consecutive strabismus following BT-A injection was neurological impairment similar to that of surgical treatment, with an incidence ranging from 0% to 25%, which is much lower than that of surgery^(5^-^10)^. A high dose of toxin, poor binocular function, and repeated injections were thought to be responsible^(5^-^7)^. In the present study, permanent consecutive strabismus was not observed in any of the patients, consistent with the findings of Cronemberger^(9)^ and Moguel^(10)^.

Secondly, this study aimed to identify the predictive factors for the successful BT-A treatment of strabismus in patients with neurological impairment. Logistic regression analysis revealed that shorter duration of strabismus and esotropic misalignment were associated with a higher treatment success rate. In addition, esotropia and lower angles of misalignment were associated with a higher success rate with a single injection. Mangan et al. also reported that a lower pretreatment angle of deviation is a predictor of success with a single injection^(12)^. Segura-Rangel et al. reported that patients with a mild cerebral injury and a lower degree of mental retardation with small-to-medium angles of esotropia had a better response, even with a history of prematurity^(6)^. Although BT-A treatment reduces the angle of deviation in patients with exotropia, it is not effective in the long term. Ameri et al. reported that a higher pre-injection deviation, younger age, severe ptosis, and lower post-injection deviation, were weakly associated with better results in patients with esotropia and CP^(7)^. They attributed this result to reinjection of patients with higher angles of deviation. They attributed this result to reinjection of patients with higher angles of deviation.

In children with neurological impairment, the treat-ment is usually delayed because of associated medical problems, unstable deviations, and unreliable measurements of the angle of misalignment before the surgery^(4)^. However, the results of the present study showed that earlier eye alignment was associated with a higher success rate. This also accords with previous observations that earlier eye alignment may lead to an earlier restoration of binocular vision and lower the risk of amblyopia in otherwise healthy children with strabismus^(17)^. Better surgical outcomes were observed in children with neurological impairment and shorter duration of strabismus^(2)^. Furthermore, an impaired balance and motor function causing postural instability have been reported in otherwise healthy children with strabismus and amblyopia^(18)^. In children with neurological impairment, strabismus correction can improve motor development, suggesting the advantage of early treatment for strabismus^(2)^.

A concern regarding early surgical treatment of children with neurological impairment is the potential risk of general anesthetic neurotoxicity in the developing brain. In animal studies, neuroapoptosis and neurodegenerative changes were demonstrated following anesthetic drug exposure^(19)^. Studies in humans were less conclusive. Some studies have suggested an association between general anesthetic exposure in early childhood and cognitive, memory, listening comprehension, and language deficits and reduced school performance^(20)^. Other studies have proposed that <1 hr of general anes-thesia in infancy does not cause significant neurocognitive or behavioral deficits^(21)^. Children with CP and HC are more likely to undergo other surgical procedures under general anesthesia, such as dental rehabilitation, orthopedic surgery, and neurosurgical procedures besides strabismus surgery. Prolonged or repeated exposures to general anesthesia during infancy may increase the risk of neurotoxicity^(22)^. The BT-A treatment offers an advantage by providing earlier eye alignment without prolonged exposure to general anesthesia, which may have harmful effects on the neural tissues of these children. In some reports, BT-A injection was performed with the “open sky” technique by conjunctival incision^(17^,^23)^. Thus, techniques that require an incision are against the non-invasive nature of BT-A treatment, which limits its repeatability, and has the disadvantage of prolonged anesthesia.

An improvement in ocular alignment following BT-A injection was observed both in the exotropic and esotropic groups; however, a less satisfactory outcome was reported in the management of exotropia than that of esotropia^(24)^. Spencer et al. attributed this difference in response to the weakness of the lateral rectus compared with the medial rectus muscle^(25)^. Segura-Rangel et al. reported that BT-A is not effective in the long term for CP with exotropia, and only one of their cases was successfully treated with BT-A injection (4.2%)^(6)^. In our study, a good outcome could be obtained in only 31.3% of patients with exotropia, which was significantly lower than the success rate in patients with esotropia. Cronemberger et al. reported that a single injection was not sufficient to achieve a successful result in the BT-A treatment of exotropia in children with neurological impairment^(9)^. Similarly, in this study, in only 18.7% of patients with exotropia, a successful alignment was obtained following a single BT-A injection.

No standard BT-A dose recommendation was established based on the angle of deviation in contrast to the standard surgical dose-response tables available for conventional strabismus surgery. In some studies, a fixed amount of toxin was used regardless of the amount of deviation, whereas in others, the BT-A dose was modified according to various factors, including the patient’s age, weight, degree of ocular deviation, and response to previous treatment^(26)^. Smaller angle of deviations were more likely to be successfully treated with BT-A than larger angles exceeding 30-35 PDs^(15)^. The effect of BT-A was shown to be dose-dependent in patients with esotropia, and increasing the dose resulted in a larger effect^(23)^. In the present study, the dosage of BT-A was adjusted according to the amount of deviation to achieve better results. Repeated BT-A dose applications might be required for large-angle deviations to improve ocular alignment^(7)^. Reinjections were required in 17 patients in the present study.

The major drawbacks of this study include the small number of patients, wide age range of the patients, absence of a control group, and absence of binocularity testing before and after BT-A injections due to young age and neuro-developmental delay of these patients. In addition, a longer follow-up of these patients including the rate of subsequent interventions can provide more definitive results.

In conclusion, in this specific group of patients with treatment challenges, BT-A injection has three major advantages. First, BT-A treatment requires less exposure to general anesthesia and lowers the risk of possible harmful effects on the neurodevelopment of the brain. Second, BT-A injection enables rapid improvement of alignment and, thus, contributes to motor development. Third, this simple and safe procedure has a low risk of consecutive deviations in children with neurological impairment, and it spares the EOM for possible further surgery. In our study, at least 60% of children could maintain eye alignment following BT-A injection; thus, surgical complications and related possible further surgeries were also avoided in the short term. The higher success rate in new-onset deviations suggested that the alignment of the eyes of children with neurological impairment should not be delayed. It will not be wrong to say that if you are not comfortable with the indication for any reason in a child with a neurological problem, you may consider using BT-A instead of delaying treat-ment. Further comparative, long-term studies with larger sample sizes should be conducted to establish the role of BT-A injection in the treatment of strabismus in children with neurological impairment.
